# Mesothelial-to-Mesenchymal Transition and Exosomes in Peritoneal Metastasis of Ovarian Cancer

**DOI:** 10.3390/ijms222111496

**Published:** 2021-10-25

**Authors:** Lucía Pascual-Antón, Beatriz Cardeñes, Ricardo Sainz de la Cuesta, Lucía González-Cortijo, Manuel López-Cabrera, Carlos Cabañas, Pilar Sandoval

**Affiliations:** 1 Tissue and Organ Homeostasis Program, Cell-Cell Communication and Inflammation Unit, Centro de Biología Molecular “Severo Ochoa” (UAM-CSIC), Consejo Superior de Investigaciones Científicas, 28049 Madrid, Spain; lucia.pascual@cbm.csic.es (L.P.-A.); bcardenes@cbm.csic.es (B.C.); mlcabrera@cbm.csic.es (M.L.-C.); 2 Department of Gynecoloy and Obstretics, Hospital Universitario Quirónsalud, 28223 Madrid, Spain; ricardo.sainz@quironsalud.es; 3 Department of Oncology, Hospital Universitario Quirónsalud, 28223 Madrid, Spain; lucia.gonzalezc@quironsalud.es; 4 Department of Immunology, Ophthalmology and Otorhinolaryngology, School of Medicine, Universidad Complutense de Madrid, 28040 Madrid, Spain; 5 Lymphocyte Immunobiology Group, Inflammatory and Immune Disorders Area, Instituto de Investigación Sanitaria Hospital 12 de Octubre (i+12), 28041 Madrid, Spain

**Keywords:** ovarian cancer, peritoneal metastasis, mesothelial-to-mesenchymal transition, exosomes

## Abstract

Most patients with ovarian cancer (OvCA) present peritoneal disseminated disease at the time of diagnosis. During peritoneal metastasis, cancer cells detach from the primary tumor and disseminate through the intraperitoneal fluid. The peritoneal mesothelial cell (PMC) monolayer that lines the abdominal cavity is the first barrier encountered by OvCA cells. Subsequent progression of tumors through the peritoneum leads to the accumulation into the peritoneal stroma of a sizeable population of carcinoma-associated fibroblasts (CAFs), which is mainly originated from a mesothelial-to-mesenchymal transition (MMT) process. A common characteristic of OvCA patients is the intraperitoneal accumulation of ascitic fluid, which is composed of cytokines, chemokines, growth factors, miRNAs, and proteins contained in exosomes, as well as tumor and mesothelial suspended cells, among other components that vary in proportion between patients. Exosomes are small extracellular vesicles that have been shown to mediate peritoneal metastasis by educating a pre-metastatic niche, promoting the accumulation of CAFs via MMT, and inducing tumor growth and chemoresistance. This review summarizes and discusses the pivotal role of exosomes and MMT as mediators of OvCA peritoneal colonization and as emerging diagnostic and therapeutic targets.

## 1. Introduction

Worldwide, 314,000 new cases of ovarian cancer (OvCA) were diagnosed in 2020, with over 207,000 disease-related deaths. OvCA is the fifth leading cause of cancer-related deaths among women, and the second one amongst gynecologic cancers (following cervical cancer) [1]. When considering industrialized countries, OvCA is the leading cause of death due to gynecological cancer. The World Health Organization (WHO) categorizes OvCA according to the origin of the cancer cell type: coelomic surface epithelial cells, stromal cells, and germ cells [2]. Epithelial OvCA (EOC), also known as ovarian carcinoma, is the most common type, accounting for over 90% of the ovarian malignancies [3]. Histologically, EOC is divided into 5 main subtypes: high-grade serous, low-grade serous, clear cell, endometrioid, and mucinous OvCA; these differ not just in their histologic features but also in their molecular characteristics, natural behavior, prognosis, and, therefore, therapeutic options [4]. High-grade serous ovarian carcinoma (HGSOC) is the most common subtype, accounting for 70–75% of EOCs. Most HGSOC patients experience non-specific symptoms, and, usually at diagnosis, the tumor presents peritoneal extension [5]. The 5-year survival rate is only 29% for these patients with clinically advanced disease [6]. Cytoreductive surgery and platinum-based chemotherapy are the keystone therapy for advanced stage OvCA [7]. However, multidrug-resistant disease is still a major problem for the overall survival of these patients, critically needing new and extending windows of therapeutic opportunities [8].

**Figure 1:**
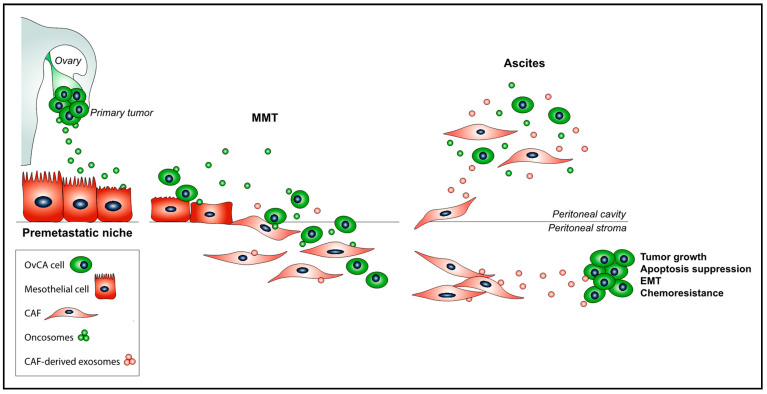
The promoting role of exosomes impinges on crucial steps of the OvCA peritoneal metastasis process: (i) Primary tumor-derived oncosomes educate a pre-metastatic peritoneal niche; (ii) during MMT, exosomes participate in the processes of adherence of OvCA cells to the mesothelium and co-invasion of OvCA cells and PMC-derived CAFs into the peritoneal stroma; and (iii) exosomes derived from CAFs induce EMT in tumor cells and suppress cancer cell apoptosis, as well as confer tumor growth and chemoresistance. Finally, OvCA cells, PMC-derived CAFs, and their, respectively, secreted exosomes are accumulated in the intraperitoneal ascitic fluid.

In contrast to other cancers, which metastasize via hematogenous or lymphatic routes, OvCA mostly disseminates intraperitoneally due to the anatomic location of the primary tumor [9]. In fact, the peritoneum is sometimes the only site of subsequent relapses, and patients invariably die due to complications derived from peritoneal disease [10]. OvCA cells detach from the primary tumor and are transported by the peritoneal fluid, where they spread by colonizing the pelvic and abdominal peritoneum [11]. The membrane that lines the abdominal cavity and all peritoneal organs is formed by a monolayer of peritoneal mesothelial cells (PMCs) with epithelial characteristics that rests on an underlying stroma composed of extracellular matrix (ECM) and connective tissue with few capillaries and resident fibroblasts [12]. The accumulation of a sizeable population of carcinoma-associated fibroblasts (CAFs), which can derive from the PMCs through a mesothelial-to-mesenchymal transition (MMT) process, is an important effect of tumor nesting in the peritoneal membrane [13,14,15]. During MMT, PMCs first dissociate from each other in the monolayer, then lose their apical-basolateral polarity, and reorganize their actin cytoskeleton to progressively acquire migratory and invasive properties [16,17]. The mesothelial cell conversion into CAFs is the result of a complex cellular reprogramming, where diverse pathways can be triggered by multiple promoting stimuli. The profibrotic transforming growth factor-β1 (TGF-β1) is considered as a prototypical inducer of MMT [18]. The receptor-mediated signaling in response to TGF-β1 can trigger the activation of a complex network of intracellular effectors, such as Smad 2/3, integrin-linked kinase (ILK), nuclear factor-𝜅B (NF-𝜅B), extracellular-signal regulated kinases 1/2 (ERKs1/2), phosphatidylinositol 3-kinase (PI3-K)/Akt pathway, c-jun-N terminal kinase (JNK), and TGF- 𝛽-activated kinase-1 (TAK-1) (reviewed in Reference [19]). On the other hand, the accumulation of large volumes of ascitic fluid in patients with OvCA peritoneal carcinomatosis has been linked to alterations of mechanical properties in the peritoneum, which, in turn, regulate the morphological and functional properties of cancer cells [20]. In fact, MMT markers overlapped with TGF-β1-dependent signaling, caveolin-1, and Yes-associated protein (YAP) activation in peritoneal biopsies of OvCA patients, supporting a cooperation between biochemical and biomechanical signal pathways in the triggering of MMT [21]. As a result of MMT, CAFs derived from PMCs synthesize ECM and secrete a variety of cytokines and growth factors that collectively promote tumor implantation, invasion, vascularization, and growth in the peritoneal stroma [13,14,15] (Figure 1).

OvCA is often accompanied by intraperitoneal accumulation of ascitic fluid, which is associated with poor prognosis [11]. Malignant ascites is the result of leakiness of microvasculature, as well as obstruction of lymphatic vessels, and is frequently a sign of peritoneal affectation [22,23,24]. Within this intraperitoneal fluidic microenvironment, tumor cells, mesothelial-derived CAFs, and infiltrating leukocytes produce a multitude of factors, including but not limited to cytokines, chemokines, and growth factors [15,23,25,26,27,28]. These autocrine and paracrine soluble molecules form complex signaling networks that govern, in part, tumor-peritoneum interactions [14]. However, large quantities of both, tumor-produced exosomes (termed “oncosomes”) and CAF-secreted exosomes, have been found in malignant ascites from OvCA patients [29]. In fact, more and more studies point to exosomes as principal mediators of tumor-stroma crosstalk and suggest that these small extracellular vesicles play an important role in favoring peritoneal metastasis, through facilitating cell adhesion, invasion, angiogenesis, proliferation, immune evasion, and chemoresistance in OvCA (reviewed in Reference [30]).

Exosomes are a subtype of 30–150-nm-sized extracellular vesicles with endocytic origin that are released to the extracellular space upon fusion of intracellular multivesicular bodies with the plasma membrane [31]. Although the content of exosomes shows specificity to the cell of origin and depends, as well, on the functional state and regulated sorting mechanisms of the cell, common components have been described for exosomes released by different cells (reviewed in Reference [32]). Typical exosomal proteins include those related to their biogenesis, such as ESCORT, ALIX, and TSG101, but also membrane proteins, such as adhesion molecules, integrins, transport and fusion proteins, heat shock proteins, cytoskeleton proteins, and the tetraspanins CD9, CD63, and CD81, which are often used as exosome detection markers [31,33,34]. In addition, exosomes are enriched in lipids, which mainly derive from the plasma membrane of the cell of origin, including cholesterol, sphingomyelin, ceramide, and phosphatidylserine (reviewed in Reference [35]). Exosomes also transport functional RNA molecules, among which are mRNAs and non-coding RNAs, such as microRNAs (miRNAs) and long non-codingRNAs (lncRNAs) [36,37]. Moreover, single and double-stranded DNA [38], as well as mitochondrial DNA, are also contained in exosomes [39,40].

Exosomes are formed via the endocytic pathway. The first step in exosome biogenesis is the formation of early endosomes through the fusion of endocytic vesicles in the cytosol. Early endosome proteins can return to the plasma membrane through recycling vesicles or, alternatively, they can mature into a specialized form of late endosomes [41], termed multivesicular bodies (MVBs), that contain intraluminal vesicles formed by the inward budding from the membrane into the lumen of MVBs [42]. The biogenesis mechanism of exosomes affects their cargo, which, in turn, determines how exosomes communicate with target cells, as well as the processes that will be regulated [43]. Three routes of exosome biogenesis have been proposed: the endosomal sorting complex for transport (ESCRT)-dependent pathway [44,45,46]; the lipid-mediated endocytosis [47,48]; and the tetraspanin-mediated mechanism [49,50,51]. Exosomes are ultimately released to the extracellular environment by the fusion of MVBs with the plasma membrane, where they can interact locally with other cells or be transported through the blood or lymph to distant sites. It has been also shown that some exosomes remain fused to the plasma membrane of the cell of origin, where they could work as signaling platforms [52,53,54].

Exosomes are important vehicles of intercellular communication through the transfer of their cargo of proteins, nucleic acids, and lipids between donor and recipient cells [55,56]. Different possibilities for the interaction between exosomes and their target cells have been proposed, including: binding of exosomes to the cell surface through ligand-receptor pairs of specific adhesion molecules; direct fusion between exosomal and cellular membranes; and internalization of exosomes into endocytic compartments through receptor-mediated endocytosis, such as the caveolin- [57] or clathrin-dependent pathways [57,58,59], through a mechanism based on lipid rafts [60], or by phagocytosis [61] or micropinocitosis [62]. The interaction with exosomes can induce direct stimulation of target cells, the transfer of membrane receptors, or the intracellular reception and integration of molecular information carried by exosomes in recipient cells.

This review focuses on providing novel insights to understand how exosomes participate in OvCA progression through the peritoneum. The new knowledge related to exosomes as potential biomarkers and therapeutic tools for peritoneal metastasis in OvCA will be also briefly discussed.

## 2. The Role of Exosomes in Ovarian Cancer Peritoneal Metastasis

Tumors originating in the abdominal cavity, such as ovarian, endometrial, pancreatic, gastric, and colorectal cancers, frequently colonize the peritoneum [9]. Interestingly, exosome-related peritoneal metastasis mechanisms have been described for these types of cancer [30,63,64]. Exosomes can be found in almost all biological fluids, including serum, saliva, urine, amniotic fluid, breast milk, and seminal fluid [65,66,67,68,69]. In recent years, the detection of exosomes in serum samples of oncological patients has raised great interest, since they have been found to play crucial roles in tumorigenesis, progression, and metastasis in different cancers that mainly disseminate through the hematogenous or lymphatic routes [70,71,72,73]. However, in the context of peritoneal metastasis, the abundance of exosomes in intra-abdominal ascitic fluid acquires a special relevance. On this note, exosomes show up to 3–4-fold increased concentrations in the malignant ascites of ovarian carcinoma patients as compared to the peritoneal fluid of non-oncological individuals [74,75]. In OvCA, exosomes exert important roles, acting directly on cancer cells, facilitating their shedding from the primary tumor, promoting their survival in the peritoneal fluid, and favoring their attachment to the PMC monolayer and subsequent invasion into the underlying peritoneal stroma [76]. Additionally, exosomes participate in the process of peritoneal metastasis by mediating complex networks of intercellular communication between OvCA cells and resident cells of the peritoneal microenvironment. In this regard, exosomes participate in the formation of a peritoneal pre-metastatic niche susceptible of being subsequently metastasized through different mechanisms, including the conversion of PMCs into CAFs via MMT, inducing immunosuppression, and promoting tumor vascularization [30] (Figure 1). On the other hand, an increasing number of studies point to exosomes as promising tools to improve OvCA outcome by reducing rates of peritoneal metastatic lesions, by facilitating early diagnosis and by interfering with tumor chemoresistance mechanisms (reviewed in References [77,78]).

## 3. Oncosomes and Their Recipient Target Cells in the Peritoneum

### 3.1. Peritoneal Mesothelial Cells

At the initial steps of peritoneal metastasis, OvCA cells directly encounter the monolayer formed by PMCs. Until recent years, it was believed that PMCs only acted as a passive mechanical barrier, avoiding tumor cell adhesion and invasion in the peritoneum and, as a consequence, preventing the formation of secondary tumor nodules into the submesothelial peritoneal stroma [79]. However, more recently, it has been reported that PMCs exert an active role in establishing a pre-metastatic niche required for the subsequent colonization of the peritoneum [80]. As in any distant metastatic process, peritoneal colonization requires the previous education of a pre-metastatic niche, a peritoneal microenvironment that favors the subsequent OvCA cell invasion through the submesothelium. PMCs are considered the principal recipient target cells for a wide range of molecules packed in oncosomes, which are initially released to the peritoneal cavity from the primary tumor site. On this note, Yokoi et al. proposed a mechanism of apoptotic PMC death via OvCA-produced extracellular vesicles carrying MMP1 mRNA [74]. Undoubtedly, the destruction of the peritoneal mesothelium barrier facilitates the establishment of metastatic implants into the peritoneal stroma. Nevertheless, in the context of peritoneal metastasis, PMCs can be converted into CAFs through an MMT process [13,14,15]. In this regard, an increasing number of reports point to oncosomes as key mediators of peritoneal metastasis through the mesenchymal reprograming of PMCs [81,82]. In fact, Wei et al. revealed the expression of specific fibroblast markers, including fibroblast activation protein (FAP) and alpha-smooth muscle actin (α-SMA), in PMCs upon in vitro and in vivo treatments with malignant ascites-derived exosomes [81].

The MMT is a consequence of a sequential process [14], and oncosome-containing proteins have been noticed to play an important role in many MMT-related steps. On this note, TGF-β1 has been found to be overexpressed in malignant ascites-derived exosomes, therefore being proposed as the principal inducer of mesenchymal conversion in the peritoneum [81]. On the other hand, the molecule CD44, a cell surface glycoprotein, has been found to be enriched in EOC-derived exosomes [82]. Interestingly, CD44 has an important role in many cellular functions, such as cell-cell interaction, adhesion, migration, and metastasis [83,84,85,86,87]. CD44 mediates tumor cell adhesion to the mesothelial monolayer through its interaction with hyaluronic acid, and, indeed, this interaction partly mediates the adhesion of OvCA cells to the peritoneal membrane [86]. In OvCA peritoneal metastasis, CD44 is transferred in oncosomes to PMCs. As a consequence, PMCs are induced to secrete MMP9, promoting ECM remodeling, clearing the mesothelial barrier, and participating in OvCA cell invasion through the peritoneal membrane [82].

In addition to proteins, ascites-isolated exosomes contain a unique miRNA signature specific to OvCA cells [88,89,90]. In this regard, it has been described that miR-99a-5p is up-regulated in oncosomes and transferred to PMCs, where, in turn, it up-regulates the expression of ECM components, such as fibronectin and vitronectin [91]. Interestingly, these two matrix proteins have been involved in OvCA cell adhesion to, and invasion through, the mesothelial monolayer that lines the peritoneal cavity [92,93]. lncRNAs have also been found to take part in OvCA progression. For example, the lncRNA SPOCD1-AS, embedded in OvCA-secreted extracellular vesicles, is transported to recipient PMCs, inducing MMT-related changes via interacting with G3BP1 protein and enhancing peritoneal colonization [94]. Besides miRNAs and lncRNAs, the exosomal circular RNA (circRNA) circPUM1 has been recently reported to participate in the peritoneal progression of OvCA. CircPUM1 can exert its tumorigenic effects by acting directly on cancer cells, but it can also be released in oncosomes and transferred to PMCs, where it up-regulates both MMP2 and NF-κB expression [95]. Zong et al. described how the circRNA circWHSC1 induces EOC metastasis by acting on the peritoneal mesothelium. CircWHSC1 is secreted by OvCA cells contained in exosomes and is taken up by PMCs, inducing up-regulation of MUC1 expression and MMT, which favors peritoneal tumor implantation [96] (Figure 1).

### 3.2. Other Oncosome-Target Cells in the Peritoneal Stroma

**Figure 2:**
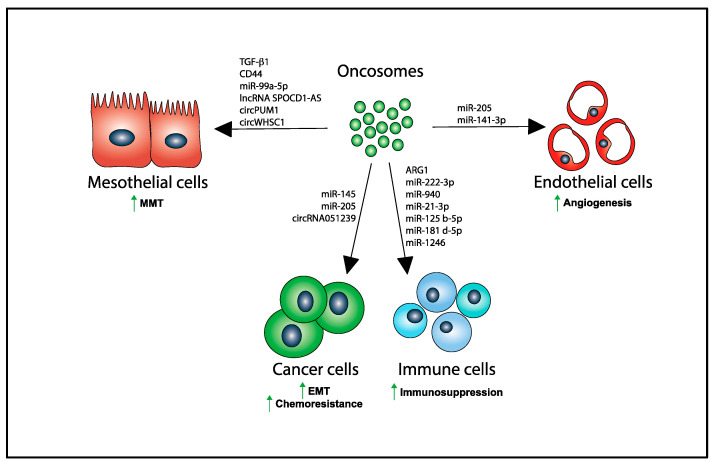
Several biomolecules are transferred via oncosomes from producing OvCA cells to their recipient target cells in the peritoneum inducing MMT in PMCs, EMT and chemoresistance in tumor cells, immunosuppression in immune cells, and angiogenesis in endothelial cells. Upward green arrows denote increase in the indicated biological process.

It is known that a crosstalk exists between tumor cells and the tumoral niche that is crucial to the development of the OvCA disease. Moreover, tumor cells can produce and release different biomolecules to their microenvironment with relevant effects on the local stroma, causing its remodeling and transformation into a pre-metastatic niche that favors ovarian tumor growth and metastasis [97,98]. Some of these biomolecules can be transferred through oncosomes from producing tumor cells to a variety of target cells, including not only the PMCs as described above but also endothelial, immune and other tumor cells, regulating gene expression and altering the phenotype and functions in these recipient cells [99,100,101,102] (Figure 2).

#### 3.2.1. Effects of Oncosomes on Immune Cells

OvCA oncosomes can exert both direct and indirect effects on innate and adaptive immune cells, promoting tumor-induced immunosuppression and evasion from immunosurveillance (reviewed in References [30,76,78]). For instance, it has been observed that they can inhibit T cell activation through their receptor (TCR) by means of different proteins expressed on their surface, including the ganglioside GD3 [103]. They can also promote apoptosis of different immune cells, including dendritic cells (DCs), peripheral blood lymphocytes, and hematopoietic stem cells, using the Fas ligand (FasL) expressed on their surface, leading to immunosuppression [104,105,106]. Another mechanism for immune suppression is the presence of arginase-1 (ARG1); these ARG1-expressing exosomes are taken up by DCs which inhibit the proliferation of CD4^+^ and CD8^+^ T-cells [107]. OvCA exosomes could also potentially down-regulate the activity of NK cells through NKG2D, similar to what has been found for other tumors [108].

Macrophages are another immune cell that plays an important role in the establishment of the pre-tumoral niche, and, in fact, they are the major type of immune cell present in the tumor environment. They can display either a pro-inflammatory (M1) or an anti-inflammatory phenotype (M2), and many studies have shown that tumor-associated macrophages are typically polarized toward an M2-like phenotype and play a crucial role in the progression of the tumor [109,110]. These cells can produce and release several molecules, such as TGFβ-1, VEGFA, IL-4, IL-5, or IL-6, that suppress the adaptive immune response and promote tumor cell survival and proliferation, invasion, and metastasis. Some studies have demonstrated that several miRNAs contained in oncosomes, such as miR-222-3p, miR-940, miR-21-3p, miR-125 b-5p, miR-181 d-5p, or miR-1246, induce the polarization toward M2 macrophages [109,110,111].

#### 3.2.2. Effects of Oncosomes on Ovarian Cancer Cells

Exosomes released by some OvCA cells can be taken up by other cancer cells, provoking changes in their phenotypic and functional properties. In this regard, the miRNA content in OvCA oncosomes has been observed to promote changes, known as epithelial-to-mesenchymal transition (EMT), in recipient tumor cells. For example, miR-145 seems to be down-regulated in OvCA oncosomes, accordingly, resulting in reduced suppression of its direct target gene CTGF (connective tissue growth factor), which is involved in tumor cell migration and adhesion [112]. A recent study shows that the amount of circRNA051239, another non-coding RNA, is increased in oncosomes and can regulate the expression of many genes in target tumor cells. This seems to have consequences in the progression of the disease by promoting cell proliferation and migration [113].

#### 3.2.3. Effects of Oncosomes on Endothelial Cells

The biomolecular cargo in OvCA oncosomes can also facilitate tumor progression by targeting, directly or indirectly, angiogenic factors. These factors can be encapsulated in exosomes and transported to endothelial cells, inducing angiogenesis and promoting metastasis [114,115]. miR-205 is one of the molecules that has been reported to be involved in the development of metastasis through the induction of tumor angiogenesis. This miRNA, which is up-regulated in OvCA patients, can promote angiogenesis by being transported to the recipient endothelial cells. On the other hand, it can also induce angiogenesis through the PTEN-AKT pathway [114]. This latter effect of miRNA-205 is thought to be mediated by targeting the VEGF pathway. Furthermore, the overexpression of miR-205 also seems to be able to promote EMT in OvCA [90]. A recent study has shown that oncosomes are also capable to up-regulate the pro-angiogenic factor VEGFA pathway in endothelial cells, driving their proliferation and migration [116].

The RNA and protein cargo in oncosomes modulate the angiogenesis process in vitro and in vivo [100]. For example, a recent study has shown that miR-141-3p in oncosomes can promote the angiogenesis process in vitro by the modulation of SOCS5, which is considered a negative regulator of the JAK-STAT and the VEGFR-2 signaling [117].

## 4. CAFs Generated via MMT Produce Exosomes That Impact on Recipient Target Ovarian Cancer Cells

While most studies are focused on oncosomes, little is known about exosomes released by cells of the surrounding tumor microenvironment and their effects in tumor progression at secondary metastatic sites.

Solid tumors are complex and unstructured organs that, in addition to cancer cells, also contain stromal cell types. It is known that CAFs represent an important population in the tumor microenvironment and participate in providing a suitable ECM and blood vessel formation to support tumor cell survival at secondary metastatic sites [118]. Furthermore, in the last few years, a number of studies have provided critical evidence regarding the significance of exosome-mediated intercellular crosstalk between CAFs and cancer cells for tumor progression [119]. For instance, in OvCA, it has been reported that CAF-derived exosomal miR-98-5p increases tumor cell proliferation and cell cycle entry, as well as confer cisplatin resistance, by targeting CDKN1A [120].

The origin of peritoneal CAFs associated with OvCA metastasis has been the subject of intense debate. However, our group demonstrated, for the first time, that an important proportion of CAFs, in peritoneal OvCA tumor implants, derives from PMCs as a consequence of an MMT process [13,14,15]. Regardless their origin, peritoneal CAFs can produce and secrete exosomes containing molecules that can be transferred, in turn, to tumor cells. On this note, it has been observed that omental CAF-derived exosomes are enriched in TGF-β1, which can be transferred to OvCA cells, triggering the acquisition of a more aggressive tumoral phenotype through undergoing EMT-related changes [121]. Interestingly, TGF-β1 has been found to be significantly up-regulated in MMT-derived CAFs isolated from the ascitic fluid of OvCA patients as compared to normal PMCs, suggesting that targeting exosomes secreted by PMCs undergoing MMT could be a potential mechanism to be interfered in the treatment of peritoneal metastasis [15]. On the other hand, Au Yeung et al. showed that miR21, a very recently identified cargo biomolecule in CAF-derived exosomes [119], is transferred from neighboring stromal cells in the omental tumor microenvironment (including CAFs and cancer-associated adipocytes) to cancer cells, where it suppresses OvCA apoptosis and confers chemoresistance by binding to its direct target APAF1 [122]. Accordingly, miR-21 has been identified as one of the most abundant miRNAs in PMCs, exhibiting mesenchymal changes upon TGF-β1 stimulation, thus providing a novel approach in the context of peritoneal carcinomatosis [123] (Figure 1).

## 5. Molecules That Mediate Specific Interactions and Uptake of Exosomes by Recipient Target Cells

As indicated above in Section 3 and Section 4, in order to act as efficient vehicles of intercellular communication, oncosomes and exosomes produced by mesothelial cells must be able to deliver their cargo of biomolecules to a variety of different target cells. The molecules that dictate these specific interactions and the subsequent uptake of exosomes by recipient cells are only beginning to be identified, and they have been reviewed by Mulcahy et al. and French et al. [124,125]. The involvement of specific molecules in these processes is frequently inferred from the use of antibodies that block exosome uptake. In this regard, different members of the integrin and immunoglobulin families of adhesion receptors were amongst the proteins consistently found to mediate these interactions, together with proteoglycans, such as CD44, and extracellular matrix proteins, such as fibronectin. Additionally, members of the tetraspanin family, such as CD9 and CD81, were also found to participate as regulators of exosome-recipient cell interactions. Our group has recently reported that the interaction of cellular integrin α5β1 with exosomal ADAM17 mediates the binding and uptake of colorectal carcinoma exosomes by recipient PMCs and cancer cells, which may bear relevance in the process of peritoneal dissemination [126]. Furthermore, exosomal tetraspanin CD9 was found to negatively regulate these interactions between cellular integrin α5β1 and its exosomal ligand ADAM17.

The question of whether cancer cells can release different subpopulations of exosomes with unique biological functions which could be targeting distinct recipient cells has not been fully resolved (reviewed in Reference [73]). However, some evidence would support that notion; for instance, neuroblastoma cells secrete different exosome populations, which differ in their cargoes and target different cells, such as neurons or glial cells [127]. Furthermore, Hoshino et al. reported, in their seminal paper, that exosomes produced by a variety of cancer cells display different integrin cargoes, which direct their selective uptake by distinct target cells, thus dictating the metastatic organotropism [52]. These findings are of upmost relevance for understanding the specific roles played by different subpopulations of cancer-produced exosomes, and, clearly, more research is still needed to further advance knowledge on this topic.

## 6. Exosomes in the Diagnosis, Prognosis, and Therapy of Ovarian Cancer Peritoneal Metastasis

The majority of women with EOC present peritoneal metastasis at the time of diagnosis. The metastatic process, however, starts long before secondary cancer implants are detected. Exosomes derived from the primary tumor prepare a cancer-favorable microenvironment in the pre-metastatic niche before the target organ is already colonized [128]. On this note, OvCA-secreted exosomes from the primary site could represent a unique opportunity to assist patients in the early detection of peritoneal dissemination. As an example, oncosomes isolated from OvCA patients carried TGF-β1, which distinguished OvCA patients from those with benign lesions [129]. Interestingly, despite their elevated TGF-β1 production, this factor has limited effects in OvCA cells, being that its contribution to peritoneal metastasis is mainly mediated through activation of Smad3-dependent TGF-β1-signaling in surrounding PMC-derived CAFs [15,130]. Moreover, high levels of oncosomal CA125 and claudin-4 have been detected in OvCA patients, significantly contributing to improved diagnosis [131]. Im et al. developed a nano-plasmonic sensor to identify oncosomes expressing CD24 and EpCAM in malignant ascites samples from OvCA patients, highlighting their potential for diagnostics [29]. Alternatively, a large battery of miRNAs has been described to be highly dysregulated in exosomes of patients with EOC [88,89,132,133,134]. Therefore, the oncosomal miRNA profiling could also be highly informative for the early diagnosis of OvCA peritoneal metastasis.

On the other hand, OvCA malignant ascites-derived exosomes display a cargo of tumor progression related proteins, such as L1CAM, CD24, ADAM10, and EMMPRIN, which have been found to correlate with worse prognosis [135]. After completion of first-line treatment, chemoresistance frequently develops, and recurrent peritoneal malignant disease is subsequently observed; this development of chemoresistance by tumor cells is a major hurdle in the treatment of OvCA. In this regard, oncosomal cargoes could also have the potential to serve as prognostic biomarkers of chemoresistance in patients with peritoneal carcinomatosis as exosomes have been proposed to play a pivotal role in the acquisition of chemotherapy resistance by OvCA cells. They have been found to mediate the acquisition of the chemoresistant phenotype in OvCA cells through multiple mechanisms, including inhibition of apoptosis, enhanced DNA repair, increased drug effluxion through the transfer of multidrug resistance (MDR) transporters, and by reducing the cellular concentration of chemotherapeutic drugs in tumoral donor cells through their expulsion in these vesicles (reviewed in References [78,136,137]. Several proteins have been found to be overexpressed in exosomes produced by chemoresistant OvCA cells, including Annexin A3 [138,139], cisplatin export transporters (MRP2 and ATP7A/B) [140], DNA methyltransferase 1 (DNMT1) [141], EpCAM [142,143], and MAGE3/6 [129]. In addition, acquired SMAD4 mutations enhance the chemoresistance profile of epithelial OvCA cells, representing a mechanism in which exchange of tumor-derived exosomes perpetuates an EMT phenotype, leading to the development of subpopulations of platinum-refractory tumor cells [144]. In addition, some miRNas have also been found to be overexpressed in OvCA tumor chemoresistance, including miR-21-3p [145], miR21 [122], miR-433 [146], miR-1246, and miR-223 [147], which could bear potential diagnostic and prognostic value for patients [145].

The singular condition of the peritoneal cavity microenvironment not only affects the chemoresistant oncosome profile but also the amount of CAF-secreted exosomes, and their cargo could be particularly relevant from a prognostic standpoint [29]. Little is known about the value of exosomes produced by MMT-derived CAFs to predict peritoneal tumor progression or therapeutic response to chemotherapy in patients with advanced OvCA. Intriguingly, Rafii et al. isolated from ascites of OvCA patients a particular type of cells with common characteristics to MCs, referred to as “Hospicells”. These cells represent a differentiated stromal subset of mesenchymal stem cells with expression of multi-drug resistance proteins. Hospicells preferentially interact with EOC cells, inducing their chemoresistance to platin and taxanes through the capture of stromal cell membrane patches by a process termed onco-trogocytosis [148]. This work led us to speculate that PMC-derived CAFs could transfer information to OvCA cells by an exosome-dependent mechanism in order to confer them a chemo-resistant phenotype. Accordingly, miR-21 is transferred in exosomes from peritoneal CAFs to cancer cells, where it suppresses OvCA apoptosis and confers chemoresistance, as it is mentioned before [122]. On this note, miR-21, known for its pro-oncogenic and pro-fibrotic activities, is highly present in OvCA-associated acites [149]. Effusion fluid-derived exosomes containing miR-21 have been associated to TGF-β-related pathways, extracellular matrix-receptor interaction, mesothelial clearance and worse prognosis value in metastatic OvCA [132]. Therefore, the detection of exosomes containing miR-21 could improve prognosis in OvCA peritoneal metastasis.

Exosomes are continuously being investigated for their applications in the therapeutic field, and, increasingly, novel options for exploiting exosomes in the treatment of OvCA peritoneal metastases are emerging [77,150]. For example, interfering with exosomal secretion or uptake mechanisms could represent an important target for therapeutical intervention. On this note, drug-resistant OvCA cells abnormally sort some lysosomal proteins showing enhanced exosomal export of cisplatin, thus this being a characteristic to be explored as a target in advanced OvCA patients [140]. Samuel et al. described that cisplatin treatment of OvCA cells led to the release of extracellular vesicles that could induce invasion and increased resistance via p38 and JNK signaling when taken up by neighboring unstressed tumor cell populations. In addition, extracellular vesicle uptake inhibitors prevented this extracellular vesicle-mediated crosstalk and, thus, sensitized cancer cells to the effects of chemotherapy [151,152]. Alternatively, removal of exosomes from malignant ascites could also contribute to improve OvCA clinical outcome. De la Fuente et al. employed exosomes purified from the ascitic fluid of OvCA patients in a murine model of peritoneal metastasis as traps to interfere with tumor cell peritoneal attachment [153]. On the other hand, interfering with the exosome-mediated MMT process could be highly advantageous in the context of peritoneal metastasis. On this note, hepatocyte growth factor (HGF) has been validated as an exosome-contained protein of interest in HGSOC patients [154]. In addition, OvCA-produced HGF is known to transform the peritoneum via MMT into a more suitable niche for subsequent tumor invasion [14,155,156]. Interestingly, siRNA against HGF packed in exosomes has been described to be transported into tumor cells metastasizing peritoneum, suppressing proliferation and migration [157]. These data lead us to speculate that exosomes delivering MMT-blocking drugs could have potential therapeutic value in OvCA peritoneal metastasis.

## 7. Conclusions

Development of peritoneal carcinomatosis is a frequent outcome in OvCA patients, which still today represents mostly a deadly incurable stage of this disease, despite the improved surgical and chemotherapeutic approaches resulting in increased progression-free disease intervals achieved in these patients over the past 30 years. A better understanding of the precise roles played by peritoneal exosomes released by tumor and stromal cells and of the mechanisms by which these extracellular vesicles deliver their biomolecular cargoes and alter the properties of recipient target cells is urgently needed. Furthermore, exosomes in OvCA are increasingly becoming recognized as key players in the conversion of PMCs into tumor-promoting CAFs through an MMT reprogramming process, which has important implications in the pathogenesis of the disease. This new knowledge on exosomes in OvCA will undoubtedly lead to the development of novel disease biomarkers, leading to earlier diagnostic procedures, and will open novel and more effective therapeutic avenues, which will collectively improve the clinical management of these women and the survival rates of this disease.

## Data Availability

Not applicable.
